# Editorial: Quantification and prediction of T-cell cross-reactivity through experimental and computational methods

**DOI:** 10.3389/fimmu.2024.1377259

**Published:** 2024-02-20

**Authors:** Dinler A. Antunes, Brian M. Baker, Markus Cornberg, Liisa K. Selin

**Affiliations:** ^1^ Department of Biology and Biochemistry, University of Houston, Houston, TX, United States; ^2^ Department of Chemistry and Biochemistry, and Harper Cancer Research Institute, University of Notre Dame, Notre Dame, IN, United States; ^3^ Department of Gastroenterology, Hepatology, Infectious Diseases and Endocrinology, Hannover Medical School, Hannover, Germany; ^4^ Centre for Individualized Infection Medicine (CiiM), c/o CRC Hannover, Hannover, Germany; ^5^ German Center for Infection Research (DZIF), Partner-site Hannover-Braunschweig, Hannover, Germany; ^6^ Department of Pathology, University of Massachusetts Medical School, Worcester, MA, United States

**Keywords:** TCR (T cell receptor), MHC, cross-reactivity, T-cell specificity, TCRpMHC, computational biology & bioinformatics

T-cell receptor (TCR) molecules play a central role in adaptive cellular immunity, enabling T-cell lymphocytes to recognize peptide-loaded Major Histocompatibility Complexes (pMHCs) at the surface of other cells (**Figure 1**). In turn, this molecular interaction can trigger T-cell activation and function (e.g., cytotoxicity or immunomodulation) (1). The specificity of the TCRpMHC interaction is essential for the efficiency of cellular immunity against infectious pathogens and cancer cells, as well as for the safety of new game-changing T-cell-based immunotherapies (2). However, it is well established that the same TCR can recognize multiple pMHC complexes with varying affinities/avidities (3–6), and such “promiscuity” has several biomedical implications, related to mechanisms of self-tolerance (1), heterologous immunity between pathogens (7, 8), autoimmunity, transplant rejection (1, 9), allergies (10), and off-target toxicity in T-cell based immunotherapies (11–13). Unfortunately, the complexity and diversity of the molecules involved in these interactions has slowed the development of both experimental and computational methods to detect, quantify, and predict these T-cell cross-reactivity events. But this picture has been changing in recent years, with exciting developments in both high-throughput experimental methods (5, 14, 15) and scalable computational approaches (16–21) for the study of TCRpMHC interactions. This Research Topic highlights recent studies in this field, including further analysis of T-cell cross-reactivity in antiviral immunity, and new computational approaches to predict TCR specificity and off-target toxicity.

**Figure 1:**
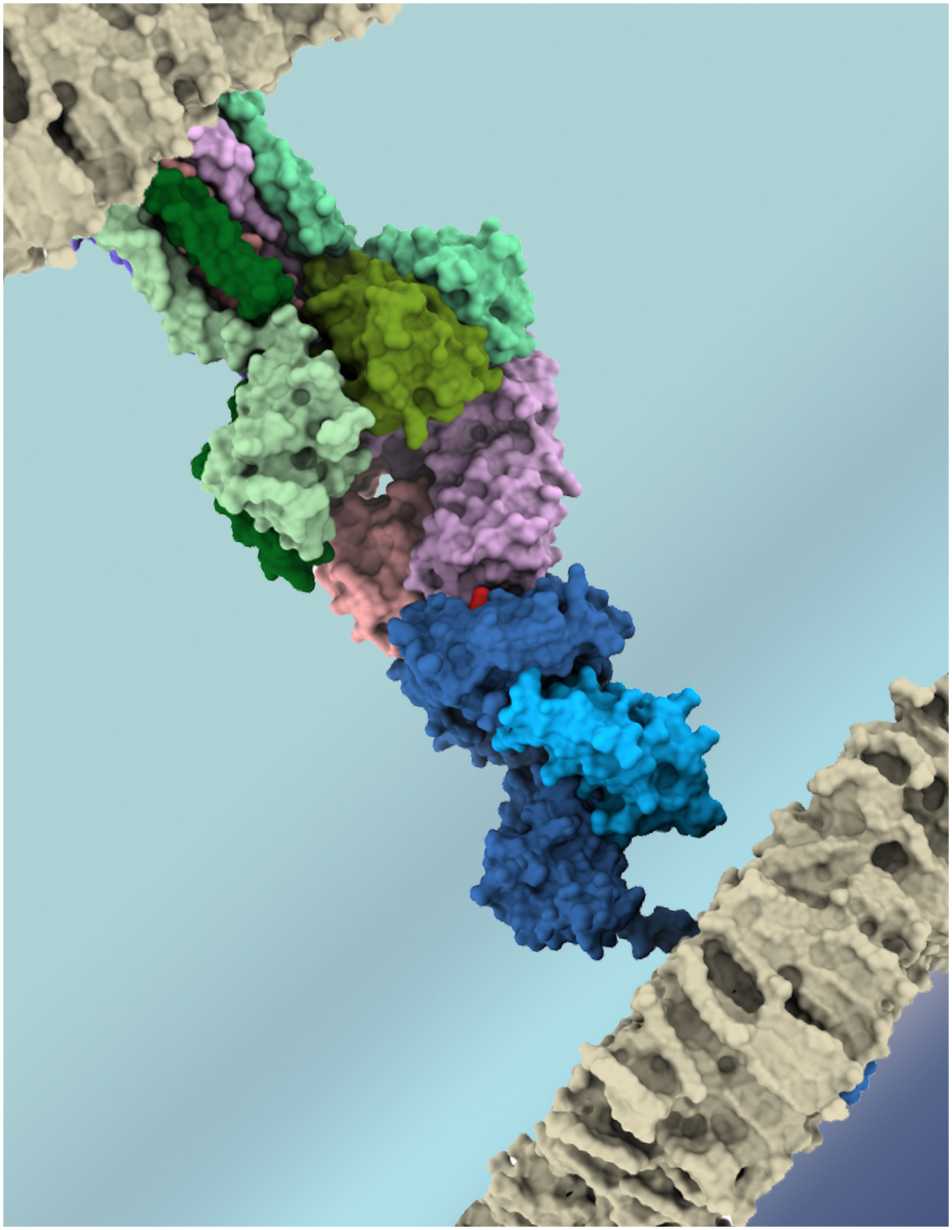
Artistic representation of the key molecules involved in T-cell recognition of an antigenic target. TCR chains (*α* and *β*) depicted in shades of purple, CD3 chains in shades of green, MHC chains (*α* and *β*2m) in shades of blue, and the peptide-target in red. Cell membranes are depicted in grey. Image obtained with UCSF ChimeraX, using PDB codes 6JXR and 5BRZ.

TCR cross-reactivity is an intrinsic feature of T-cell biology, required to maximize immunity against an overwhelming diversity of antigenic peptide-targets (4, 13, 22, 23). The benefits of cross-reactivity for antiviral immunity are further evidenced by Petrova et al. They analyzed the recall CD8+ T-cell response to variants of the well-characterized Influenza A M1_58−66_ peptide, to show how cross-reactive T-cells can be selected by naturally occurring non-infective variants or quasispecies of RNA viruses. Since immunological history also affects the selection of these cross-reactive cells, Tarabini et al. leveraged existing immunoinformatics methods to investigate the potential link between BCG vaccination and reduced severity of COVID-19 cases. Building upon previous work on image-based analysis of modeled pMHC complexes (6, 24–26), they screened over 13.5 million possible cross-reactive peptide pairs from BCG and SARS-CoV-2, identifying multiple high-density “neighborhoods” of cross-reactive peptides which could be driving heterologous immunity induced by BCG vaccination. Similarly, Antonio et al. used pMHC structural modeling to investigate how previous infections may produce heterologous immunity in a global scale, therefore mitigating the full lethal potential of the COVID-19 pandemic. They identified similar structural fingerprints across peptides derived from other coronaviruses and from unrelated viruses involved in endemic human infections, which could trigger cross-reactive T-cell responses against SARS-CoV-2 variants. In fact, cross-reactivities with SARS-CoV-2-derived peptides have been since reported in multiple studies (27, 28).

These efforts to leverage pMHC structural data represent a more recent trend among computational methods to interpret or predict T-cell cross-reactivity, as previously reviewed by others (6, 29, 30). Attesting to the fast progress in the field, our Research Topic includes three additional computational tools for cross-reactivity prediction guided by structural data. First, Mendes et al. leveraged the analysis of electrostatic potentials over the TCR-interacting surfaces of pMHC complexes to develop MatchTope. The tool relies on a modified version of PIPSA (31) to enable the structure-based clustering of similar pMHC structures, which are in turn more likely to be recognized by the same TCR. Second, Hall-Swan et al. implemented PepSim, a webserver for T-cell cross-reactivity prediction based on a novel similarity score for pMHC structures. PepSim represents the pMHC solvent-accessible surface as a triangular mesh, which is then annotated with biochemical features, including electrostatic potential, hydrophobicity, and hydrogen bond potential. Finally, Fonseca and Antunes introduced CrossDome, an R-based tool to predict the risk for off-target toxicity in T-cell-based immunotherapy. By default, the tool performs a sequence-based peptide-centered search for biochemically similar off-targets, leveraging publicly available multiomics data from healthy tissues (e.g., immunopeptidomics and gene expression data). However, the authors also demonstrate how structural data of TCRpMHC complexes can be used to perform a TCR-centered prediction, enabling to refine the list of putative off-targets for a specific T-cell clone. This is an important direction for future development, considering the greater availability of TCR sequences (e.g., single-cell TCRseq), and the growing interest in analyzing TCR specificity across T-cell repertoires (32). For now, analyses at that scale are mostly limited to sequence-based methods, as reviewed by Ghoreyshi and George. But the authors also describe the emergence of hybrid quantitative computational approaches for studying TCR specificity, with emphasis on the growing role of deep learning architectures behind these methods.

This Research Topic highlights a transition to a new age in the study of T-cell cross-reactivity, in which AI-powered structure-guided computational prediction of TCR specificity for polyclonal T-cell repertoires, and high-throughput experimental validation of T-cell activation, will be used to guide the development of better and safer vaccines and T-cell-based immunotherapies (13, 33). However, there are still challenges ahead, as we try to incorporate in these scalable computational methods a more refined understanding of the forces and dynamics driving the affinity, avidity and specificity of TCRpMHC interactions (34, 35).

## Author contributions

DA: Conceptualization, Supervision, Writing – original draft, Writing – review & editing. BB: Writing – review & editing. MC: Writing – review & editing. LS: Writing – review & editing.
